# Novel cytomegalovirus variants in immunocompromised hosts: genetic insights and clinical significance

**DOI:** 10.3389/fmicb.2025.1677054

**Published:** 2025-11-04

**Authors:** Abdulrahman M. Alsweed, Madian S. Alsanea, Reem S. Almaghrabi, Ahmed A. Al-Qahtani, Mohammad AlSuhaibani, Sami H. Alhajjar, Fatimah S. Alhamlan

**Affiliations:** ^1^Department of Pediatrics, Section of Infectious Disease, Pediatric and Women’s Centre of Excellence, King Faisal Specialist Hospital and Research Centre, Riyadh, Saudi Arabia; ^2^Department of Infection and Immunity, King Faisal Specialist Hospital and Research Centre, Riyadh, Saudi Arabia; ^3^Organ Transplant Center of Excellence, King Faisal Specialist Hospital and Research Centre, Riyadh, Saudi Arabia; ^4^College of Medicine, Alfaisal University, Riyadh, Saudi Arabia

**Keywords:** herpesviridae, cytomegalovirus, antiviral therapy, immunocompromised, drug resistance mutation

## Abstract

**Background:**

Human cytomegalovirus (HCMV) is a significant opportunistic pathogen affecting immunocompromised individuals, particularly solid organ and hematopoietic stem cell transplant recipients. The emergence of mutations within conserved genomic regions of HCMV genes targeted by antiviral therapies, significantly complicating the interpretation of resistance and treatment decisions. Although the molecular characterization of such mutations and their clinical correlation are critical to guide appropriate therapeutic strategies, the significance of many detected mutations and variants, even those in conserved regions, remain uncertain in terms of *in vitro* or *in vivo* drug resistance. In this study, we clinically evaluated 15 such novel mutations.

**Methods:**

Clinical specimens from immunocompromised and transplant patients with confirmed HCMV DNAemia were sequenced for *UL97*, *UL54*, and *UL56*. The detected variants were aligned with the HCMV Merlin reference genome and evaluated for novelty and conservation. Patient records were retrospectively reviewed to assess antiviral regimens, virological responses, and clinical outcomes.

**Results:**

In total, 13 patients (25%) exhibited novel *UL97*, *UL54*, and *UL56* mutations. Four patients (30.77%) met the criteria for refractory HCMV DNAemia with varying clinical responses. Some patients responded to first-line antiviral agents despite carrying resistance-associated variants. Notably, the *G579C* mutation in *UL97* and *A835T* mutation in *UL54* were found within conserved domains crucial for kinase and polymerase functions, indicating their potential functional significance. One patient carried the established *UL54 P522S* mutation, which has been associated with intermediate ganciclovir resistance. Two cases of severe immunosuppression and persistent viremia led to mortality, demonstrating the impact of host immunity on treatment response.

**Conclusion:**

Interpreting cytomegalovirus (HCMV) drug resistance mutations requires a comprehensive approach that integrates molecular data with clinical context. Early genotypic analysis can guide antiviral therapy; however, improved classification of mutations based on predicted resistance potential and phenotypic characteristics may optimize clinical decision-making. These insights emphasize the need for personalized management strategies in immunocompromised patients.

## 1 Introduction

Human Cytomegalovirus (HCMV) is considered the largest human herpesvirus. The wild-type variant was characterized by sequencing the 235,645 base-pair genome of the low-passage strain Merlin (Gatherer et al., 2011). HCMV infection remains a substantial cause of morbidity in immunocompromised populations, particularly among solid organ and bone marrow transplant recipients (Kotton et al., 2018). Advances in molecular diagnostics have yielded deep insights into HCMV genetic polymorphisms and facilitated mutation detection associated with antiviral resistance. Although recombinant phenotyping is widely used for antiviral resistance analysis (Chou et al., 2005), various emerging phenomena, including cross-resistance mutations, development of drug resistant mutations (DRMs) during antiviral therapy, and clinically refractory HCMV DNAemia with no known DRMs, while some uncharacterized variants were detected (Chou, 2020).

In this study, we describe the comprehensive assessment of a series of clinical cases involving HCMV variants with known resistance mutations and uncharacterized genetic variants, many of which exhibited responsiveness to standard first-line antiviral therapy. Our findings highlight the challenges in interpreting genotypic and recombinant phenotypic data and reinforce the importance of integrating molecular findings with clinical outcomes. This study sought to correlate clinical variability with known genotypic resistance, improve understanding of viral behavior among different host factors that significantly affect HCMV infection management, and assess the potential impact of novel mutations.

## 2 Materials and methods

### 2.1 Ethics statement

The research was conducted in compliance with institutional policies and national guidelines for studies involving human subjects. Ethical approval was obtained from the Research Advisory Council (RAC) at King Faisal Specialist Hospital and Research Centre in Saudi Arabia (RAC #2230035). Because this was a retrospective study that analyzed anonymized clinical samples, the Research Ethics Committee waived the requirement for informed consent. All research procedures adhered to the ethical standards outlined in the Declaration of Helsinki.

### 2.2 Clinical samples

A total of 52 plasma samples, collected between 2022 and 2025 at King Faisal Specialist Hospital and Research Center, were used to develop the in-house assay. All reported HCMV levels are expressed in international units per milliliter (IU/mL) by RT-PCR technology using an Abbott Alinity m instrument, which detects and quantifies genotypes (gB1-gB4) with a range of 30 to 100,000,000 IU/mL. A result below 30 indicates HCMV viral load detected but not accurately quantified. All clinical data, including viral load measurements, were systematically extracted from the hospital’s electronic medical records. The viral loads of the included plasma samples ranged from 740 to 2,379,966 IU/mL. All patients were receiving antiviral treatment at the time of sample collection. The antiviral agents administered at the institution include ganciclovir, valganciclovir, and foscarnet, with letermovir administered in selected cases.

To optimize DNA quality for sequencing, plasma samples were carefully handled and aliquoted to preserve sample integrity and support possible retesting. Plasma was used instead of whole blood to align with standard protocols for viral DNA quantification. The leftover plasma samples, previously tested for CMV in the hospital laboratory, were stored at −80 °C. These Samples were excluded if they were not stored at 2 °C–4 °C for up to 4 days or at −70 °C or lower, if labeling or documentation was incomplete, if the sample type was incorrect, or if the volume was insufficient for testing. All samples were successfully amplified and sequenced.

### 2.3 Novel mutation detection

Primers designed in-house were used to amplify genes with drug-resistant mutations, including *UL97*, *UL56*, and *UL54*. The recommended regions for genotyping antiviral resistance mutations are codons 335–665 for UL97, 252–999 for UL54, and 230–370 for UL56 (Kotton et al., 2018). However, in our in-house assay, we expanded the amplified regions to cover codons 325–670 for UL97, 207–1,120 for UL54, and 185–450 for UL56. This adjustment was made to mitigate the poor sequence quality frequently observed near the start of sequencing reads, corresponding to primer binding sites. By extending the target regions beyond guideline recommendations, we ensured higher quality sequencing data, thereby enhancing the accuracy of mutation detection and overall assay performance. Comprehensive details of the original in-house assay development and validation, including primer design, amplification parameters, and validation procedures, are described in a separate manuscript by Alsanea et al. (2025, currently under review). Following amplification, the amplicons were sequenced at the Sequencing Core Facility, Department of Genetics, King Faisal Specialist Hospital and Research Centre using an ABI3730XL DNA Analyzer (Applied Biosystems, Foster City, CA, United States). The DNASTAR Lasergene 15.0 package (SeqMan Pro, version 15; DNASTAR, Inc., Madison, WI, United States) was used to validate the chromatogram files, clean the sequences, and assemble the contigs using human herpesvirus 5 strain Merlin genome (NC_006273.2) as a reference sequence.

All contigs were translated into amino acid sequences using EditSeq version 15. These sequences were then aligned with the reference sequences *UL97* (YP_081544.1), *UL54* (YP_081513.1), and *UL56* (YP_081515.1) using the MUSCLE tool to identify the amino acid mutations. A comprehensive database of all reported and published mutations facilitated the categorization of these mutations, as outlined in the literature (Chou, 2020). Amino acid substitutions were classified into three groups: confirmed drug resistance mutations, natural polymorphisms, and novel mutations detected for the first time in our population. Eventually, to locate the nucleic acid alterations that caused the amino acid changes, (NC_006273.2) was used to align the nucleic acid sequences using ClustalOmega on MegAlign Pro version 15.

### 2.4 Confirmation protocol for novel mutations

**Figure 1:**
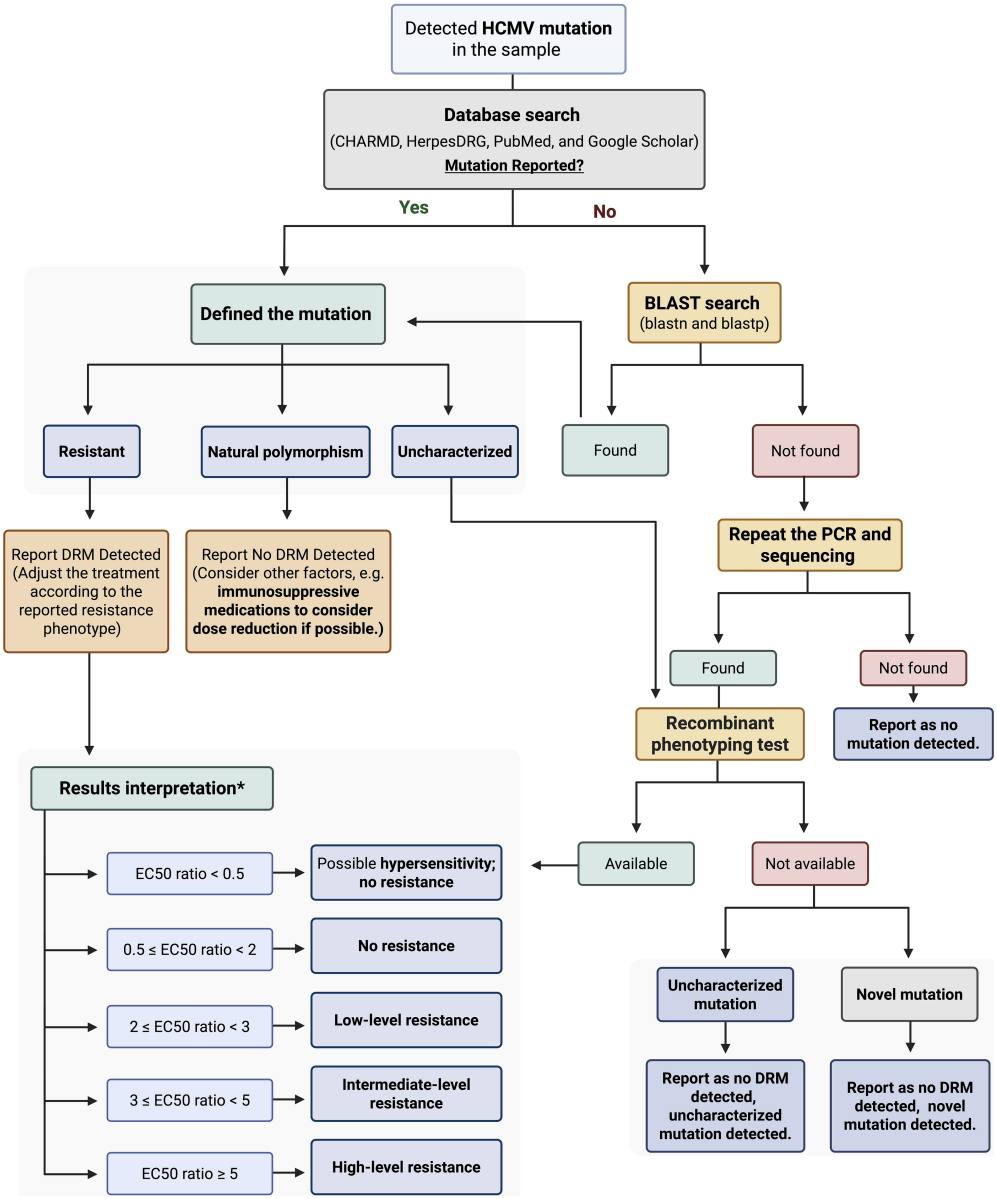
Workflow for classification, confirmation, and interpretation of detected HCMV mutations. *The results interpretation was explained in Comprehensive Herpesviruses Antiviral drug Resistance Mutation Database (CHARMD). HCMV: Human Cytomegalovirus; BLAST: Basic Local Alignment Search Tool; DRM: Drug Resistant Mutation; PCR: Polymerase Chain Reaction; EC₅₀ Half Maximal Effective Concentration. Created with BioRender.com.

We evaluated HCMV mutations using the approach outlined in Figure 1, which summarizes the key steps for detecting and analyzing mutations. This approach allowed for systematic identification and comparison of variant profiles across samples. Literature searches were conducted on the PubMed and Google Scholar databases up to May 2025, using the following search terms individually or in combination: “HCMV mutations,” “*UL97* mutations,” “*UL56* mutations,” “*UL54* mutations,” “drug-resistant HCMV mutations,” “HCMV and transplantation,” “HCMV management,” and “HCMV treatment.” Only articles published in English were considered. Each presumed novel mutation was also included as a search term. All mutations that were not documented in the literature were considered for subsequent analysis. Additionally, two databases were used to search for mutations: the Comprehensive Herpesviruses Antiviral Drug Resistance Mutation Database (CHARMD) (Tilloy et al., 2024) and HerpesDRG (Charles et al., 2024). Each sample underwent two independent rounds of amplification. If identical mutations were detected in both reactions, the validity of the finding was confirmed, and the PCR-induced error was excluded.

Novel mutations were validated by individually testing the nucleic and amino acid sequences using different Basic Local Alignment Search Tool (BLAST) tools.^1^ BLASTp was utilized to align the amino acid sequences with all publicly available sequences across four databases: UniProtKB/Swiss-Prot (RefSSwissProt), reference proteins (RefSeq protein), Patented protein sequences (patina), and non-redundant protein sequences (nr) (Table 1). BLASTn was used to align the nucleic acid sequences with all published sequences in three databases: Core Nucleotide Database (core_nt), Nucleotide Collection (nr/nt), and Patent Sequences (pat) (Table 2).

**Table 1:** All detected mutations in the study population and the corresponding search results for the amino acid sequences.

Gene	Patient ID	Sample number	Mutation	The percentage of highest similarity in each database							
				swissprot	Acc	refseq protein	Acc	pataa	Acc	nr	Acc
UL97	11	197	G579C	99.70%	P16788.1	99.70%	YP_081544.1	99.70%	CAC42715.1	99.70%	AKI09486.1
UL54	7	74	T252M	99.56%	Q6SW77.1	99.56%	YP_081513.1	99.45%	AAS31232.1	99.78%	QPI35313.1
	5	29	G267S	99.31%	P08546.2	99.31%	YP_081513.1	99.31%	AAS31232.1	99.77%	AAD30069.1
	3	27	D346N	99.54%	P08546.2	99.54%	YP_081513.1	99.54%	AAS31232.1	99.77%	AAD30086.1
	2	7	N618D	99.41%	P08546.2	99.41%	YP_081513.1	99.41%	AAS31232.1	99.88%	AMJ52924.1
	13	56	M637V	99.55%	P08546.2	99.55%	YP_081513.1	99.55%	AAS31232.1	99.85%	AFR55047.1
	9	76	P656L	99.08%	P08546.2	99.08%	YP_081513.1	99.08%	AAO93934.1	99.54%	AKI11465.1
	9	76	G672D	99.08%	P08546.2	99.08%	YP_081513.1	99.08%	AAO93934.1	99.54%	AKI11465.1
	10	24	G680D	99.28%	Q6SW77.1	99.28%	YP_081513.1	99.28%	AAO93934.1	99.76%	AKI21156.1
	12	206	A835T	99.08%	P08546.2	99.08%	YP_081513.1	99.08%	AAS31232.1	99.65%	CAG7582472.1
	1	3	E858K	99.29%	P08546.2	99.29%	YP_081513.1	99.29%	AAS31232.1	99.76%	AHJ84974.1
	7	74	E882G	99.56%	Q6SW77.1	99.56%	YP_081513.1	99.45%	AAS31232.1	99.78%	QPI35313.1
UL56	6	53	D239N	99.59%	P16724.1	99.18%	YP_081515.1	99.59%	AGV78716.1	99.59%	ANQ47130.1
	8	68	L373H	99.62%	P16724.1	99.24%	YP_081515.1	99.62%	AGV78716.1	99.62%	AKI26056.1
	4 and 5	28	E424K	99.58%	P16724.1	99.16%	YP_081515.1	99.58%	UOK99462.1	99.58%	APA46230.1

RefSSwissProt, UniProtKB/Swiss-Prot; RefSeq protein, Reference proteins; patina, Patented protein sequences; nr, non-redundant protein sequences.

**Table 2:** All detected mutations in the study population and the corresponding search results for the nucleotide sequences.

Gene	Patient ID	Sample number	Mutation	Highest similarity percentage in each database					
				Core-nt	Acc	nr/nt	Acc	pat	Acc
UL97	11	197	*G1735T*	99.80%	HQ158774.1	99.80%	KR534213.1	99.60%	MY904332.1
UL54	7	74	*C755T*	99.53%	KR534200.1	99.53%	KR534200.1	99.34%	MY904329.1
	5	29	*G799A*	99.81%	KC519320.1	99.81%	KY490078.1	99.61%	MV974705.1
	3	27	*G1036A*	99.31%	AF133609.1	99.31%	AF133609.1	99.04%	MY904331.1
	2	7	*A1852G*	99.96%	KR534204.1	99.96%	KR534204.1	99.80%	MV974705.1
	13	56	*A1909G*	99.80%	JX512206.1	99.80%	JX512206.1	99.21	MV974705.1
	9	76	*C1967T*	99.24%	KR534200.1	99.24%	KR534200.1	98.63%	MY904331.1
	9	76	*G2015A*	99.24%	KR534200.1	99.24%	KR534200.1	98.63%	MY904331.1
	10	24	*G2039A*	98.96%	KP745714.1	98.96%	MN075802.1	98.88%	MY904333.1
	12	206	*G2503A*	99.38%	KR534202.1	99.38%	PQ867562.1	98.96%	MX631210.1
	1	3	*G2572A*	99.92%	KR534206.1	99.92%	KR534206.1	99.8%	MV974705.1
	7	74	*A2645G*	99.53%	KR534200.1	99.53%	KR534200.1	99.34%	MY904329.1
UL56	6	53	*G715A*	99.73%	KP745722.1	99.73%	MT044480.1	99.73%	PL313345.1
	8	68	*T1118A*	99.87%	KP745648.1	99.87%	MT044480.1	99.87%	PL313345.1
	4 and 5	28	*G1270A*	99.86%	KP745719.1	99.86%	MT044480.1	99.86%	PL313345.1

Core_nt, Core Nucleotide Database; nr/nt, Nucleotide Collection; pat, Patent Sequences.

Similarity scores were determined to identify the most closely matching sequence for alignment with the sample sequence, confirming that the dissimilar mutation was indeed the novel mutation. MegAlign Pro version 15 was used to perform the alignment, the MUSCLE tool to align the amino acid sequences, and the ClustalOmega tool to align the nucleic acid sequences.

## 3 Results

This study included only patients with novel mutations. The viral loads of the samples ranged from 2,439 IU/mL to 127,478 IU/mL. The analyzed cases exhibited known natural polymorphisms, previously reported uncharacterized mutations, or drug resistance mutations. Notably, 15 mutations, 11 in UL54, 1 in UL97, and 3 in UL56, were identified as novel, having not been previously reported as polymorphisms, uncharacterized variants, or drug resistance–associated mutations.

Comparative sequence analysis against multiple reference databases showed that the sample sequences were not identical to previously reported sequences. The highest similarity score for UL97 was 99.70% at the amino acid level and 99.80% at the nucleic acid level. The maximum similarity for UL54 was 99.88% for amino acid sequences and 99.96% for nucleic acid sequences. For UL56, the highest similarity reached 99.62% at the amino acid level and 99.87% at the nucleic acid level. Tables 1, 2 provide details of the novel mutations, similarity scores, and references databases.

**Figure 2:**
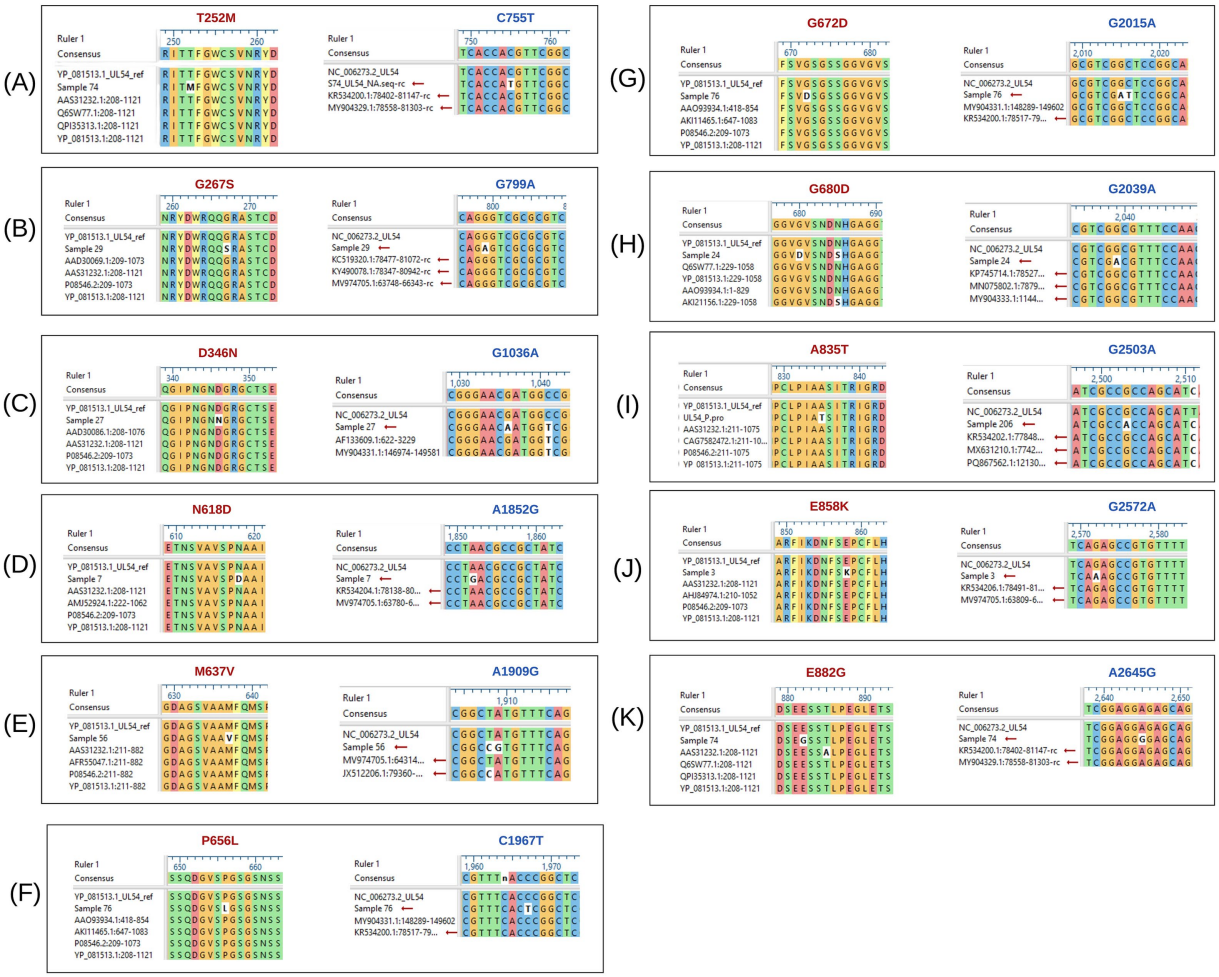
Multiple sequence alignment of *UL54* nucleotide and amino acid sequences, highlighting detected mutations. The black boxes labeled **(A)** to **(K)** depict the alignment of *UL54* sequences. Mutations shown in red represent amino acid changes, while those in blue indicate nucleotide-level mutations.

**Figure 3:**
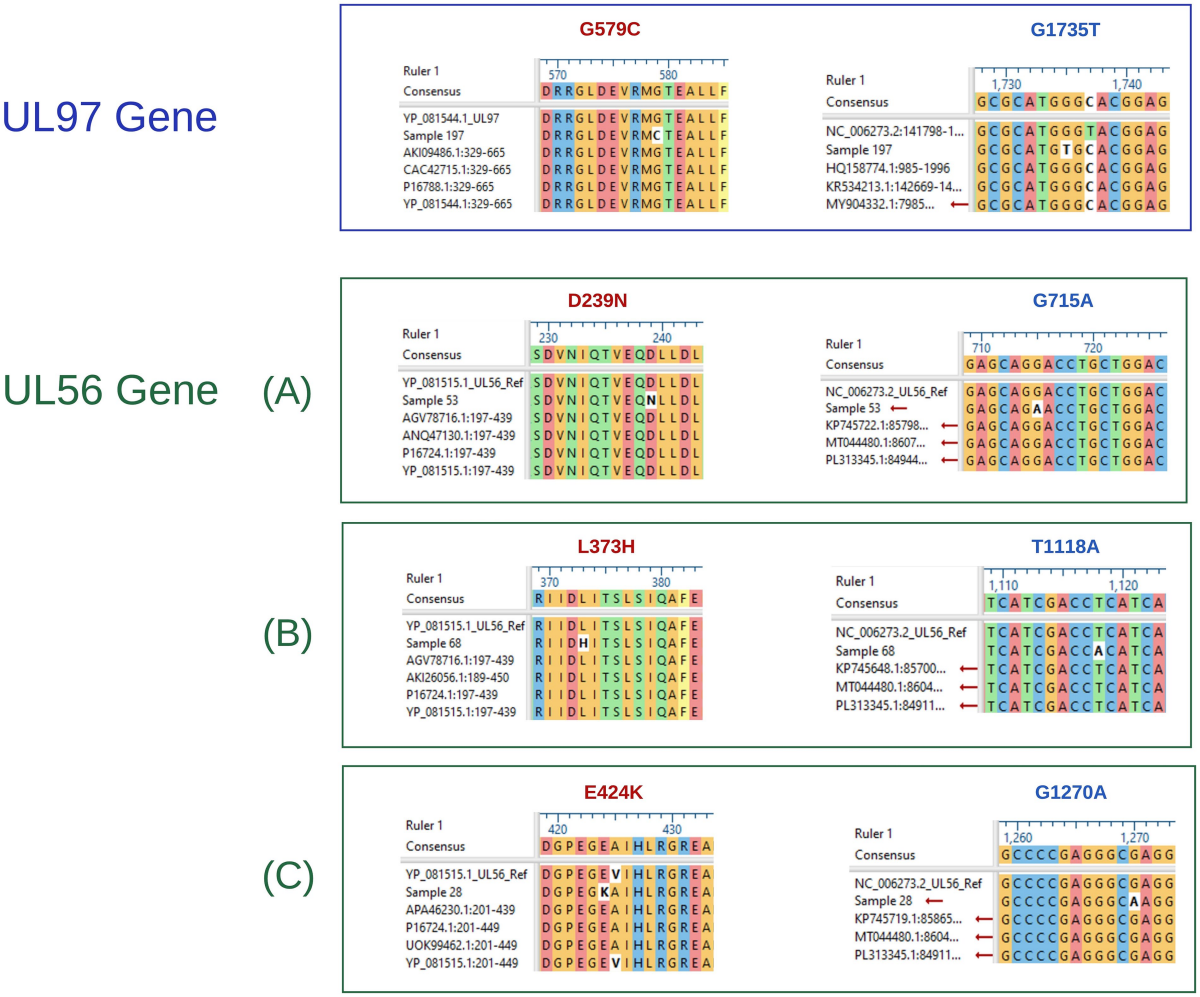
*UL97*, and *UL56* nucleotide and amino acid aligned sequences, showing detected mutations. The blue box shows the aligned *UL97* sequence, whereas the green boxes labeled **(A–C)** represent the aligned *UL56* sequences. Within all groups, mutations written in red indicate amino acid alterations, whereas those in blue correspond to nucleotide mutations.

**Figure 4:**
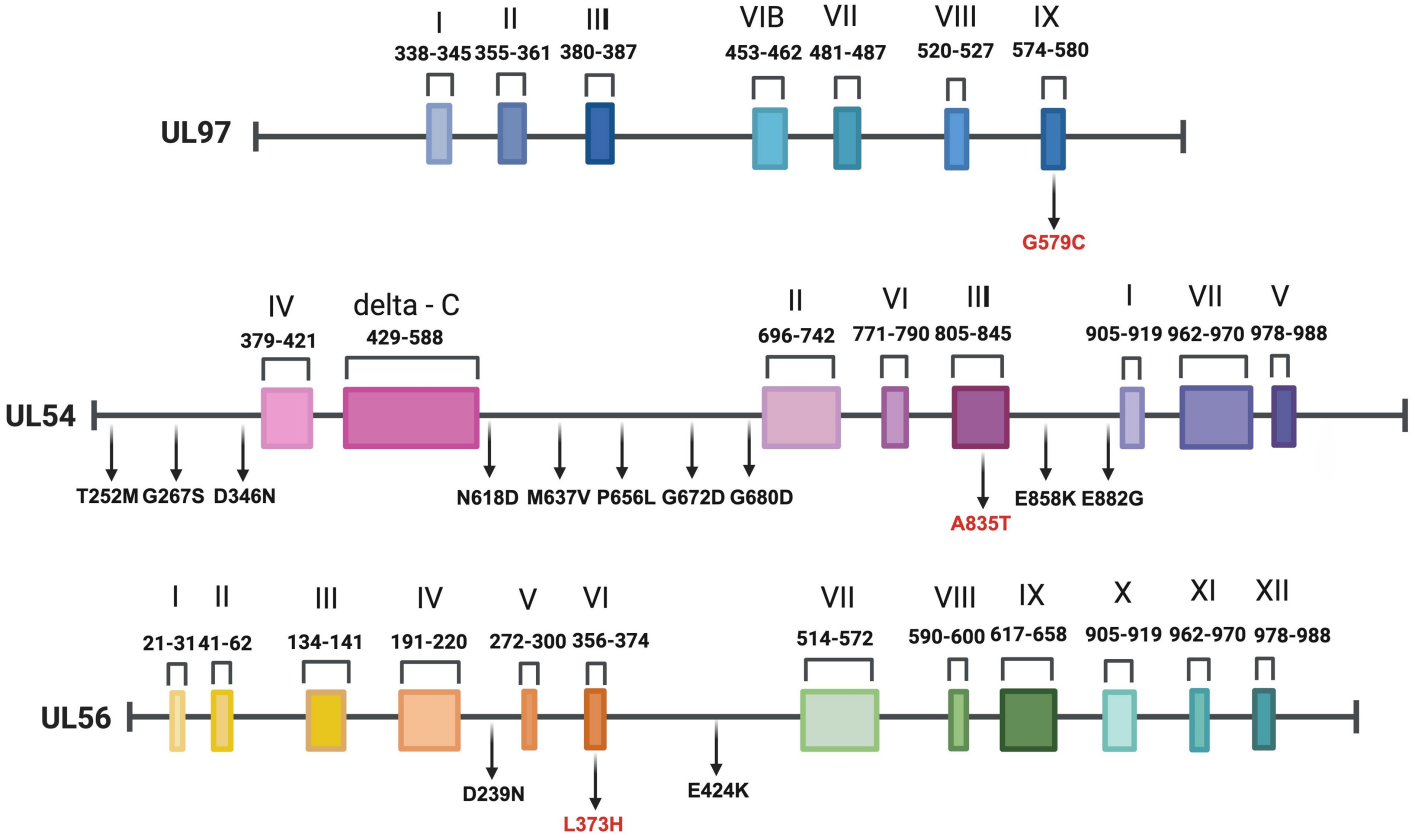
Gene organization and mutation mapping of *UL97*, *UL54*, and *UL56* illustrating positions of amino acid variants detected in this study. We identified one UL97, 11 UL54, and three UL56 novel mutations for the first time in our population. Mutations located within conserved regions are written in red. Created with BioRender.com.

Furthermore, none of the confirmed mutations in UL54 (T252M, G267S, D346N, N618D, M637V, P656L, G672D, G680D, A835T, E858K, and E882G) were identified in both CHARMD and HerpesDRG databases. All the novel mutations are shown in Figure 2, while the confirmed mutations in UL97 (G579C) and UL56 (D239N, L373H, and E424K) are shown in Figure 3. Some mutations were linked to highly variable regions, whereas others occurred in known conserved regions, as illustrated in Figure 4. The clinical outcomes and DNAemia clearance patterns are detailed in Table 3.

**Table 3:** Cases with novel HCMV mutations.

Patient ID	Case	Mutation(s) detected	Host factor	HCMV serostatus	Refractory HCMV DNAemia	Treatment response	Outcome
1	1	*UL56: V425A* *UL54: S655L, N685S, E858K, L897S, N898D, and G1031C*	Liver Transplant	R + D+	No	Responded to IV GCV then VALG	DNAemia resolved
2	2	*UL54: N618D, S655L, L897S, and N898D*	Bone Marrow Transplantation (Matched Related Donor)	R + D+	No	Responded to GCV, then FOS	DNAemia resolved
3	3	*UL97: A427T* *UL56: V425A* *UL54: D346N, S675G, L897S, and N898D*	Bone Marrow Transplant (Haploidentical)	R + D+	Yes	Partial response to combined GCV, FOS, and HCMV-IgG	The patient died
4	4	*UL97: A427T* *UL56: E424K, V425A, and M442T* *UL54: G874R, L897S, and N898D*	Bone Marrow Transplant (Haploidentical)	R + D+	Yes	Responded to FOS	DNAemia resolved
5	5	*UL56: E424K, V425A, and M442T* *UL54: G267S, **P522S^**, S655L, N685S, L897S, and N898D*	Immunodeficiency (CVID)	-	Yes	VALG treatment, sudden uncontrolled DNAemia	The patient died
6	6	*UL56: D239N, V425A*	Renal Transplant	R − D+	No	Responded to GCV and then VALG	DNAemia resolved
7	7	*UL56: V425A* *UL54: T252M, E882G, N898D, and A1108T*	Liver Transplant	R − D+	No	Adjusted GCV dosing and VALG	DNAemia resolved
8	8	*UL56: L373H, V425A*	Liver Transplantation (Deceased Donor)	R + D+	No	Responded to GCV and then VALG	DNAemia resolved
9	9	*UL56: V425A* *UL54: P656L, G672D, L897S, and N898D*	Liver Transplantation (Living Donor)	R − D+	No	Responded to the GCV	DNAemia resolved
10	10	*UL56: V355A and V425A* *UL54: S655L, G680D, N685S, L897S, and N898D*	Heart Transplant	R − D+	Yes	Initially refractory to GCV responded to FOS and HCMV-IG, then to GCV and maintained.	DNAemia resolved
11	11	*UL97: G579C* *UL56: V425A* *UL54: N898D*	Bilateral Lung Transplantation	R + D+	No	Responded to the VALG	DNAemia resolved
12	12	*UL97: A427T* *UL56: missed region* *UL54: S655L, N685S, E793G, A835T, L897S, and N898D*	Bone Marrow Transplantation (Matched Related Donor)	R + D+	No	Responded to the VALG	DNAemia resolved
13	13	*UL56: V425A* *UL54: S655L, M637V, and N685S*	Renal Transplant (Living related)	R + D+	No	Responded to GCV and then VALG	DNAemia resolved

*Refractory HCMV infection: Increase by > 1 log10 HCMV DNA levels in blood or plasma after at least 2 weeks of appropriate anti-HCMV medication. HCMV: human cytomegalovirus; GCV: ganciclovir; VALG: valganciclovir; FOS: foscarnet; R: recipient of transplanted organ/bone marrow, D: donor, (CVID) common variable immunodeficiency. Reported mutations are natural polymorphisms except marked mutations; bold indicates a known drug resistance mutation; red indicates a novel mutation. ^IC_50_ for GCV was reported as 3.1 (intermediate level) (Cihlar, T et al. Ref. 5).

### 3.1 Refractory CMV DNAemia (*n* = 4)

Refractory CMV DNAemia was defined as either an increase of more than 1 log₁₀ in CMV DNA levels from the peak viral load, or persistence of viral load with ≤1 log₁₀ increase or decrease after at least 2 weeks of appropriate antiviral therapy. Four patients met this criterion and are described below:

*Case 3*: A baby boy, product of a vaginal delivery after an uneventful pregnancy, was transferred at 5 weeks of age for evaluation for hematopoietic stem cell transplantation (HSCT) due to a family history of severe combined immunodeficiency (SCID), Omenn phenotype. Since the first week of life, he experienced recurrent infections, including gastroenteritis, pneumonia, and skin rash. Upon transfer, he developed sepsis and septic shock. Blood culture grew methicillin-resistant *Staphylococcus aureus* (MRSA), and he was treated accordingly with vancomycin. Cerebrospinal fluid studies were negative for bacterial and viral pathogens. On 31 December 2023 (at 6 weeks of age), HCMV DNAemia was detected at 23,104 IU/mL. Ganciclovir induction was initiated on January 1, 2024; however, DNAemia rose to 127,478 IU/mL on January 7. Resistance testing at this point identified no known resistance mutations but revealed novel variants. DNAemia further increased to 158,391 IU/mL by January 14, prompting a switch to foscarnet on January 11. Despite therapy, DNAemia peaked at 1,456,557 IU/mL on 21 January. Ophthalmology evaluation showed no retinitis. Pulmonary imaging raised concern for pneumonitis, but bronchoalveolar lavage was not feasible, and therefore HCMV-related disease could not be confirmed. Dual antiviral therapy and adjunctive HCMV-specific immunoglobulin were provided empirically. No HLA-matched donor was available; therefore, bridging therapy continued. DNAemia subsequently decreased, reaching 14,258 IU/mL on 28 January and 342 IU/mL on 10 March. The patient proceeded to a mismatched transplant on 21 March while maintained on dual antivirals. DNAemia reached its nadir at 66 IU/mL on 7 April 2024. All subsequent blood cultures were negative. Shortly thereafter, he developed febrile neutropenia, severe pneumonitis, acute respiratory distress syndrome (ARDS) complicated by pulmonary hemorrhage, and died on 13 April 2024 (see Table 4).

**Table 4:** Patient characteristics, viral load, antiviral therapy, and immunosuppression.

ID	Sample number	Age/Gender	Co-infections	Viral load (IU/mL)	Sample collection date	Anti-viral Therapy						Immuno-suppressants
						GCV/VALG	FOS	CDV	MAR	LET	HCMV IG	
1	3	19y/F	N/A	11,532	03/11/2022	Yes	No	No	No	No	No	Steroids, FK, MMF
2	7	14 m/M	Bacterial Sepsis	4,119	02/02/2023	Yes	Yes	No	No	No	No	CSA
3	27	5w/M	Bacteremia/Pneumonia	127,478	07/01/2024	Yes	Yes	No	No	No	Yes	N/A
4	28	16 m/M	Bacterial sepsis	21,611	10/03/2024	Yes	Yes	No	No	No	No	PTCY, MMF, CSA
5	29	42y/M	Pneumonia	2,439	24/03/2024	Yes	No	No	No	No	No	N/A
6	53	14y/F	Bacterial Sepsis	2,566	28/07/2024	Yes	No	No	No	No	No	Steroids, AZA, FK
7	74	2y/M	C diff colitis, SSTI	11,955	06/01/2025	Yes	No	No	No	No	No	Steroids, FK
8	68	28y/M	N/A	19,461	25/11/2024	Yes	No	No	No	No	No	Steroids, FK, MMF
9	76	14 m/F	N/A	5,069	26/01/2025	Yes	No	No	No	No	No	Steroids, FK, MMF
10	24	47y/M	N/A	36,867	28/09/2023	Yes	Yes	No	No	No	Yes	Steroids, MMF, SIR
11	197	17y/M	N/A	48,427	26/09/2023	Yes	No	No	No	No	No	Steroids, FK, MMF
12	206	18y/M	N/A	6,931	21/04/2024	Yes	No	No	No	No	No	FK
13	56	21y/F	N/A	7,425	05/08/2024	Yes	No	No	No	No	No	Steroids, FK, MMF

FK: Tacrolimus (FK506); MMF: Mycophenolate Mofetil; CSA: Cyclosporine A; PTCY: Post-Transplant Cyclophosphamide; AZA: Azathioprine; SIR: Sirolimus (Rapamycin); GCV: Ganciclovir; FOS: Foscarnet; VALG: Valganciclovir; CDV: Cidofovir; HCMV IG: Human Cytomegalovirus Immunoglobulin; MAR: Maribavir; LET: Letermovir; *C. difficile*: Clostridioides difficile; SSTI: Skin and Soft Tissue Infection; N/A: Not Applicable.

Outcome: Death due to severe pneumonitis with ARDS and pulmonary hemorrhage (HCMV disease could not be confirmed).

*Case 4*: A 16-month-old boy with SCID underwent haploidentical stem cell transplantation on 18 January 2024 using Treosulfan, Fludarabine, Anti-thymocyte globulin (ATG), and Thiotepa conditioning. GVHD prophylaxis included post-transplant cyclophosphamide (PTCY), mycophenolate mofetil (MMF), and cyclosporine A. He was readmitted on 25 February 2024 with an Extended-Spectrum Beta-Lactamase (ESBL) *Klebsiella pneumoniae* central line–associated bloodstream infection and concurrent parainfluenza pneumonia. During this episode, HCMV DNAemia was first detected at 187 IU/mL, and ganciclovir was initiated. By 3 March, DNAemia was 981 IU/mL, and by 10 March, it had risen more than 1 log to 21,611 IU/mL. At that time, foscarnet was initiated. Under this regimen, DNAemia trended down, reaching low levels by 28 April. Therapy was transitioned to oral valganciclovir after consecutive results of 34 IU/mL and <30 IU/mL over 2 weeks (3 June). DNAemia became undetectable on 16 June and remained negative during subsequent monitoring. His last available result on 5 January 2025 confirmed no detectable HCMV DNAemia.

Outcome: Survived, with clearance of HCMV DNAemia, last follow-up July 2025 with no CMV DNAemia.

*Case 5*: A 42-year-old man with common variable immunodeficiency (CVID) and T-cell dysfunction had multiple comorbidities, including bronchiectasis, *Mycobacterium abscessus* lung colonization, chronic sinusitis, bilateral hearing loss, and recurrent gastrointestinal infections (Campylobacter, *E. coli*). He had recurrent HCMV DNAemia since 2017, managed with valganciclovir 900 mg twice daily, with intermittent low-level rises but no documented end-organ disease. In March 2024, DNAemia was 2,439 IU/mL (prior level 523 IU/mL 4 months earlier). Resistance testing was requested at that time. In April 2024, he was diagnosed with hepatic angiosarcoma and opted for palliative care with a Do Not Attempt Resuscitation (DNAR) code status. He was admitted on 3 May 2024 with pneumonia; DNAemia at admission was 196,278 IU/mL. His clinical condition deteriorated, and he died on 8 May 2024.

Outcome: Death due to progressive pneumonia in the setting of underlying immunodeficiency and high-level HCMV DNAemia.

*Case 10*: A 47-year-old man with non-ischemic cardiomyopathy underwent orthotopic cardiac transplantation on 14 July 2023. His immunosuppression included steroids, MMF, and sirolimus. His post-transplant course was complicated by pneumonia and bacteremia with *Serratia marcescens*, for which he completed 2 weeks of meropenem with repeated negative blood cultures. On 10 September 2023, while on valganciclovir prophylaxis, HCMV DNAemia was detected at 6,621 IU/mL. He was admitted and started on ganciclovir induction therapy. Despite treatment, DNAemia rose. He developed worsening cardiac function (reduced left and right ventricular function), prompting an endomyocardial biopsy, which demonstrated acute cellular rejection but no HCMV cytopathic changes. He was provided steroids, ATG, and plasmapheresis. Therapy was switched to foscarnet, and he received three doses of HCMV immunoglobulin. After 2 weeks of foscarnet, and with no resistance mutations identified, ganciclovir was reintroduced. DNAemia gradually decreased and became undetectable on 20 November 2023.

Outcome: Survived, with resolution of HCMV DNAemia, last follow-up July 2025 with no CMV DNAemia.

## 4 Discussion

HCMV genotypic resistance is evolving rapidly as more data emerge, particularly those from immunocompromised and transplant patient populations. This analysis of 13 cases provides further insight into the clinical implications of novel and previously uncharacterized mutations in the HCMV DNA polymerase (*UL54*), kinase (*UL97*), and terminase (*UL56*) genes.

Consistent with previous studies, most patients were solid organ or hematopoietic stem cell transplant recipients or were otherwise immunocompromised. This population has a well-established increased risk of HCMV reactivation and antiviral resistance development (Gilbert and Boivin, 2005; Lurain and Chou, 2010). Although genotypic analysis provides rapid and sensitive detection by enabling direct testing on clinical specimens and circumventing the need for viral culture, it is fundamentally unable to distinguish true resistance mutations from natural sequence polymorphisms without phenotypic confirmation (Gilbert and Boivin, 2005; Komatsu et al., 2014).

Many identified mutations did not correspond to clinical antiviral resistance or refractory DNAemia. Furthermore, most patients responded well to first-line antivirals, such as ganciclovir (GCV) or valganciclovir (VALG), achieving HCMV DNAemia clearance without therapy modification. Thus, many of these mutations may be benign natural polymorphisms rather than true resistance-associated variants.

This observation aligns with prior findings. Lurain and Chou (2010) noted that polymorphic variations in *UL54* are relatively common and do not always correlate with clinical resistance, mainly when mutations occur outside of established functional domains critical for antiviral binding or enzymatic activity. Similarly, naturally occurring polymorphisms must be differentiated from clinically significant resistance mutations to avoid unnecessary therapy changes, as (Sahoo et al., 2013) emphasized.

However, few patients, particularly those with severe immunosuppression, experienced more complicated disease courses, demonstrating that host immune status remains a dominant determinant of HCMV clinical outcomes, often even more so than viral genotype alone. This supports the findings from many cohorts, especially in stem cell transplant settings, which demonstrated that immune reconstitution is critical for controlling HCMV infection, independent of antiviral therapy (Gallez-Hawkins et al., 2005; Gratama et al., 2008).

One patient (Case 5) had the *UL54 P522S* mutation in our series, a variant previously described as conferring intermediate resistance to GCV (Chou, 2020). This confirms that while novel mutations are prominent, previously recognized resistance mutations are still clinically significant when they occur. The concurrence of the known *UL54 P522S* mutation with novel mutations complicates the study of resistance, as the effects of the novel mutations may be masked or altered by the established resistance mechanism, causing difficulty in isolating their individual impacts on antiviral treatment. Recombinant phenotyping of this novel mutation (*G267S*) may help distinguish its antiviral resistance effect.

Our study faces some challenges. The number of samples analyzed is limited because there are few cases with suspected drug resistance. Additionally, we could not perform the phenotyping test due to technical complexity. Therefore, we emphasize the importance of conducting phenotypic assays, which directly measure antiviral susceptibility *in vitro*, to properly assess the functional impact of these new mutations. Phenotyping is particularly essential when addressing mutations located within conserved regions of the UL54 DNA polymerase, UL97 kinase, or UL56 terminase complex, as they could affect drug binding or enzyme activity, even if not previously documented.

## 5 Conclusion

HCMV drug resistance mutations must be analyzed cautiously because the host response can be the main determining factor for DNAemia clearance. Early and specific DRM reporting is crucial, especially in immunocompromised hosts, for which genotyping is the best modality. However, we recommend interpreting these findings according to the clinical response and known recombinant phenotypic testing methods (EC50/IC50). The additional benefit of classifying DRMs as low, intermediate, or high can facilitate clinical decisions on class switching and dose adjustments of ongoing antiviral therapy, particularly in the initial treatment response. Interestingly, the persistence of DNAemia may indicate antiviral resistance, even in the absence of detectable resistance mutations, as observed in case 10. While our study provides important insights into HCMV novel mutations and their potential clinical significance, a larger cohort with more clinical samples is necessary to confirm these findings. Additionally, phenotypic resistance testing is crucial for validating the functional impact of these mutations and for further understanding their role in antiviral resistance.

## Data Availability

The datasets presented in this study can be found in online repositories. The names of the repository/repositories and accession number(s) can be found at: https://www.ncbi.nlm.nih.gov/genbank/, PX048028; https://www.ncbi.nlm.nih.gov/genbank/, PX048029; https://www.ncbi.nlm.nih.gov/genbank/, PX048030; https://www.ncbi.nlm.nih.gov/genbank/, PX048031; https://www.ncbi.nlm.nih.gov/genbank/, PX048032; https://www.ncbi.nlm.nih.gov/genbank/, PX048033; https://www.ncbi.nlm.nih.gov/genbank/, PX048034; https://www.ncbi.nlm.nih.gov/genbank/, PX048035; https://www.ncbi.nlm.nih.gov/genbank/, PX048036; https://www.ncbi.nlm.nih.gov/genbank/, PX048037; https://www.ncbi.nlm.nih.gov/genbank/, PX048038; https://www.ncbi.nlm.nih.gov/genbank/, PX048039; https://www.ncbi.nlm.nih.gov/genbank/, PX048040.

## Footnotes

^1^ https://blast.ncbi.nlm.nih.gov/Blast.cgi
